# Rising Demand for Winter Crops Under Climate Change: Breeding for Winter Hardiness in Autumn-Sown Legumes

**DOI:** 10.3390/life16010017

**Published:** 2025-12-22

**Authors:** Katalin Magyar-Tábori, Sripada M. Udupa, Alexandra Hanász, Csaba Juhász, Nóra Mendler-Drienyovszki

**Affiliations:** 1 Department of Crop Production, Applied Ecology and Plant Breeding, Faculty of Agricultural and Food Sciences and Environmental Management, University of Debrecen, 4032 Debrecen, Hungary; mtaborik@agr.unideb.hu; 2 International Center for Agricultural Research in the Dry Areas (ICARDA), P.O. Box 6299, Rabat 10112, Morocco; s.udupa@cgiar.org; 3 Research Institute of Nyíregyháza, Institutes for Agricultural Research and Educational Farm (IAREF), University of Debrecen, P.O. Box 12, 4400 Nyiregyhaza, Hungary; juhasz.csaba@agr.unideb.hu (C.J.); mendlerne@agr.unideb.hu (N.M.-D.)

**Keywords:** autumn sowing, overwintering, cool-season legumes, breeding, climate adaptation

## Abstract

Climate change in the Pannonian region is accelerating a shift toward autumn sowing of cool-season grain legumes (pea, faba bean, lentil, chickpea, lupine) to achieve higher yields, greater biomass production, enhanced nitrogen fixation, improved soil cover, and superior resource use efficiency compared with spring sowing. However, successful overwintering depends on the availability of robust winter-hardy cultivars. This review synthesizes recent breeding advances, integrating traditional approaches—such as germplasm screening, hybridization, and field-based selection—with genomics-assisted strategies, including genome-wide association studies (GWAS), quantitative trait locus (QTL) mapping, marker-assisted selection (MAS), and CRISPR/Cas-mediated editing of CBF transcription factors. Key physiological mechanisms—LT_50_ determination, cold acclimation, osmoprotectant accumulation (sugars, proline), and membrane stability—are assessed using field survival rates, electrolyte leakage assays, and chlorophyll fluorescence measurements. Despite challenges posed by genotype × environment interactions, variable winter severity, and polygenic trait control, the release of cultivars worldwide (e.g., ‘NS-Mraz’, ‘Lavinia F’, ‘Ghab series’, ‘Pinklevi’, and ‘Rézi’) and ongoing breeding programs demonstrate substantial progress. Future breeding efforts will increasingly rely on genomic selection (GS), high-throughput phenomics, pangenomics, and G×E modeling to accelerate the development of climate-resilient legume cultivars, ensuring stable and sustainable production under increasingly unpredictable winter conditions.

## 1. Introduction

One of the most pressing environmental challenges facing humanity today is climate change. Since the late 19th century, the global mean surface temperature has increased by approximately 1.18 °C, with the rate of warming accelerating markedly over the past four decades [1]. According to NASA analysis (2024), 2023 was the warmest year on record, with global temperatures reaching approximately 1.4 °C above pre-industrial levels [2]. There is a broad scientific consensus that climate change is primarily driven by human activities, particularly those altering the composition of the atmosphere. The enhanced greenhouse effect resulting from elevated concentrations of greenhouse gases, especially carbon dioxide (CO_2_), is the principal cause of ongoing global warming [3,4].

The effects of global warming are expected to last for a long time and have many—mainly negative—consequences. In addition to climate change, it will be accompanied by fluctuating temperatures, increased flood and storm damage, melting ice caps, rising sea levels, impaired biodiversity, more frequent epidemics, and higher death rates. The frequency of extreme weather incidents increases, e.g., heavy rains, heat waves, floods, severe storms, droughts, and wildfires [5]. Global warming does not affect different regions, biomes, and living communities in the same way; the warming trend will be greater at higher latitudes. Moisture and temperature maxima shift north; the temperate and boreal forests will be more affected than those in tropical and subtropical areas. In addition, flora will be more affected than fauna [5]. However, the effects of climate change can be not only negative but also positive [6]; e.g., the changes in plant phenology and the shifting of the limits of cultivation can even lead to higher yields [7].

Climate change represents one of the most significant global challenges to agricultural sustainability and food security. The agricultural sector is highly sensitive to climatic variability, including changes in temperature, precipitation, and the frequency of extreme weather events, which directly influence crop yields, livestock productivity, and ecosystem stability [8]. The increase in air temperature occurring during climate change can result in significant changes in the phenological stages of plants; this is especially true for temperate plant species. The growth period can be extended both by earlier sprouting in spring and by a longer vegetation period in autumn, as has been reported for several woody plants [9,10]. In addition, the climatic limits of certain crop production systems are shifting toward the North [7,11].

Since many affected growing areas are characterized by an increase in air temperature and, with it, a decrease in the number of frosty days [7], strategies for adapting to climate change include changing the time of cultivation [12]. In addition, it becomes necessary to introduce new species and varieties showing good adaptability into cultivation practice [6,11]. Cultivating new varieties with better stress tolerance, together with an adapted sowing time, can be one of the best responses to the challenges induced by climate change [11]. In Central Europe, increasing the sowing area of autumn-sown leguminous crops can be a reasonable response to changes in agro-climatic conditions [13].

The climatic changes characteristic of the Central European Pannonian region include, in addition to typically higher temperatures throughout the year, a decrease in the amount of precipitation. An unfavorable change in the distribution of precipitation means a significant decrease in the summer precipitation [14]. However, due to the significant temperature fluctuations characteristic of the region, the lack of snow cover, and the occurrence of late spring frosts, the overwintering of autumn-sown crops can still be risky; therefore, it is necessary to develop new plant varieties with good winter hardiness [15].

Under autumn sowing, cold damage in plants is a major constraint, which can be caused by low temperatures that are above freezing (i.e., chilling, which occurs between 0 and 15 °C) and below freezing (0 °C) levels [16].

As global temperatures rise and winter seasons become milder, the necessity for breeding winter-hardy legumes becomes increasingly critical. An effective way to mitigate the effects of climate change (especially in Central Europe) could be to introduce autumn sowing instead of spring sowing for certain crops [17]. The sustainability of legume crops in changing climates hinges on their ability to withstand colder temperatures and extreme weather patterns. Breeding winter-hardy legumes ensures that farmers can maintain productivity and food security despite these challenges. Autumn sowing is mainly important for cool-season grain legumes, such as peas, faba beans, lentils, chickpeas, and lupines [18]. Additionally, forage, green manure, and cover crop legumes are also often sown in autumn [19].

Winter-hardy legumes exhibit significant variances in frost tolerance, primarily influenced by genetic factors and environmental conditions. Research indicates that specific genetic traits associated with frost tolerance in pea cultivars can be effectively harnessed through marker-assisted selection, enhancing breeding efficiency for winter hardiness [20]. Furthermore, breeding efforts focusing on increased winter hardiness are vital in regions facing extreme winter conditions, particularly where average minimum soil temperatures can drop below −10 °C [21]. Such winter adaptability is especially crucial in temperate regions where cold stress can severely impact legume yield [22].

Moreover, incorporating winter-hardy germplasm into breeding programs significantly improves the chances of successful autumn sowing [23]. The genetic variations observed in winter lentils and alfalfa populations further highlight the potential for breeding programs to enhance these traits across different legume species [24,25]. For instance, breeding winter-hardy lentils can ensure survival through harsh winters, essential for cultivation in colder climates [26]. In regions such as the continental Balkans, breeding efforts have yielded promising advancements in developing cold-tolerant annual legumes. The successful introduction of winter-hardy varieties can lead to increased legume production, which addresses food security issues linked to climate change [27]. Evaluation methods that combine controlled environmental assessments with field trials provide deeper insights into the genetic foundations of winter hardiness, bolstering the effectiveness of breeding programs [28]. As climate models project an increase in winter temperatures, the adaptation of legumes through breeding programs also involves ensuring their successful integration into existing agricultural practices. Therefore, advancing the genetic profile of legumes for winter hardiness should be prioritized in agricultural research and development strategies to mitigate the impacts of climate change on crop production.

This review synthesizes recent studies on breeding for overwintering capacity in cool-season grain legumes, focusing on objectives, methods, and outcomes to guide future cultivar development under changing climatic conditions.

## 2. Agronomic and Ecological Analysis of Autumn-Sown Plants

Investigating the agronomic and economic benefits, risks, and costs associated with autumn sowing reveals a complex interplay of agronomic factors and economic implications.

### 2.1. Agronomic and Ecological Benefits of Autumn-Sown Plants

Autumn-sown crops have several agronomic and ecological advantages over spring-sown varieties. These advantages stem from phenological timing (early root and leaf development, vernalization), improved soil and water use, and positive interactions between soil protection and plant physiological performance. Early emergence and vernalization provide a longer vegetation period, which can improve yield and spring growth dynamism [29]. Autumn sowing promotes deeper root development, increases water and nutrient utilization, and reduces drought sensitivity [30]. Winter mulching of the soil reduces erosion and nutrient leaching, thereby improving soil structure and organic matter content. In addition, autumn-emerged crops often suppress weeds more effectively and, in some cases, also show better quality parameters (e.g., protein content) [31]. Autumn sowing generally improves yield, biomass, and resource use efficiency across cereals and industrial crops compared to spring sowing (Table 1). However, the success of autumn sowing depends on the choice of variety, local climatic conditions, and the appropriate sowing time.

In the case of autumn-sown crops, such as winter wheat and winter rye, it is obvious that good winter tolerance is an important breeding goal, although choosing the proper sowing date is also a key factor in successful crop production [32,33]. The development of new genotypes with adequate winter hardiness is suggested to be based on the multiple natural selections for survival under field conditions in the case of winter wheat [34]. Moreover, in addition to tolerance to low temperatures and freezing, several other factors may affect the winter hardiness, such as duration of the acclimation, humidity, pests, and pathogens; even in the case of perennial plants, weather characteristics during the previous summer can play a role [35].

Most cereals are sown in autumn, but there are also spring varieties and facultative genotypes that can be sown in both periods [29,36]. Genotypes can differ significantly in their frost tolerance, and the winter hardiness of varieties is also influenced by their geographical origin and year of registration [37]. Facultative triticale cultivars were tested under temperate conditions in Austria for autumn (October) and spring (March) sowing. Although autumn-sown facultative triticale developed more slowly, it could be harvested earlier and produced higher aboveground dry matter yields than spring-sown crops. They also showed greater tolerance to dry conditions than spring-sown crops, and ‘Trimmer’ was proven to be more tolerant to unfavorable growing conditions compared to ‘Agrano’ [38]. Similarly, the yield and energy output of facultative wheat (*Triticum aestivum* L., ‘Lennox’) were much higher when sown in autumn (October) than when sown in spring (March) [39]. The yield components (grains per ear, grains per m^2^, and thousand-grain weight) of facultative wheat were higher when it was sown in autumn compared to those sown in spring. However, outputs were in interaction with nitrogen fertilization, and the extent of the differences depended on the nitrogen dose [40]. In intercropping experiments conducted in Austria, it was observed that autumn sowing was more beneficial for both facultative wheat and facultative peas: the plants developed faster, grew taller, and had higher aboveground dry matter production [29]. In the case of traditionally spring-sown oats, Chinese trial results suggest that ‘WC109’ is a suitable genotype for widespread autumn sowing in Southwest China, enabling very cost-effective production in rice fields [41].

Autumn sowing may also be beneficial for industrial crops that are usually sown in spring. Higher seed yield was harvested when poppy (*Papaver somniferum* L.) was sown in autumn instead of spring in Austrian experiments [42]. Although the alkaloid and oil content were higher in yield harvested from plots sown in spring in experiments in Turkey, due to higher capsule yield, the total obtained morphine yield was higher on plots sown in autumn [43]. Similarly, autumn sowing of safflower (*Carthamus tinctorius* L.) in Greece resulted in higher yields of both achene and oil [44]. Sugar beet can be grown by autumn sowing in warmer regions, including southern Europe (Spain) (*Beta vulgaris* L.). Due to the increasing water shortage in the growing areas, autumn sowing of sugar beet may also be justified in cooler regions [45]. However, problems may arise during autumn cultivation, such as the occurrence of bolting, the risk of frost damage, and lower yields compared to spring-sown sugar beets [45]. Lower sugar content of the root was also observed [46]. Moreover, significant differences were found, for example, in the yield and sugar content of sugar beets sown in autumn at different sowing times, while the dates of spring sowing did not result in significant differences [47]. However, in experiments performed in Germany, about 26% higher yield could be predicted due to increased light absorption for sugar beet (*Beta vulgaris* L.) sown in autumn compared to those sown in spring [48].

Experiments have been conducted with several other species, which may be sown both in spring and in autumn, such as camelina (*Camelina sativa* (L.) Crantz) [49] and linseed (*Linum usitatissimus* L.) [50].

**Table 1:** Summary of autumn sowing benefits across crop types.

Crop	Key Benefits of Autumn Sowing	Location	Reference
Triticale	produced higher aboveground dry matter yield, earlier harvest, greater tolerance to drought	Austria	[38]
Facultative Wheat	yield, energy output, grains ear^−1^, grains m^−2^, thousand-grain weight	Austria	[39,40]
Facultative Wheat + Pea (intercropping)	faster development, grew taller, higher aboveground dry matter production	Austria	[29]
Oats	better yield, cost-efficiency (in rice fields)	China	[41]
Poppy	higher seed and morphine yield	Austria and Turkey	[42,43]
Safflower	achene and oil yield	Greece	[44]
Sugar beet	higher yield (26%)	Germany	[48]
Camelina, Linseed	yield potential	Serbia and Italy	[49,50]

### 2.2. Agronomic and Ecological Risks of Autumn-Sown Plants

Although the potential agronomic advantages of autumn-sown legumes can be significant, the switch to autumn-sown can also present several disadvantages, the economic implications of which can be significant. We did not plan to perform a comprehensive economic analysis in this study, but some important factors determining cost-effectiveness should be highlighted.

Timing of sowing significantly influences yield and survival rates of various legumes in Mediterranean climates, where inappropriate autumn sowing can lead to increased mortality rates due to frost and chilling [51]. This indicates that while autumn sowing can offer benefits such as longer vegetative growth periods, risks of severe winter conditions must also be accounted for, thus necessitating insurance coverage for potential crop losses due to winterkill [52]. Additionally, economic considerations regarding seed prices are significant, particularly for winter-hardy varieties. The price of seed represents a significant proportion of the production cost. If the production of winter-hardy varieties is more expensive, this will also be reflected in the price of the seed. In fact, these seeds often come at higher prices, justified by their capacity to mitigate risks associated with yield loss due to winterkill (Table 2). Research focusing on winter crops shows that while cultivating winter-hardy varieties can improve yield and profitability, they come with higher initial costs [51,53].

The data shows that the conditions of seed production, e.g., whether it was done conventionally or organically, determine the price of the seed. There is a difference between varieties that can be sown in autumn (winter type) and those that are sown in spring. However, the cost of seed is not only influenced by the price of the seed but also by the amount of seed required. In the case of autumn sowing, it may be necessary to use up to 30% more seed, but in the case of a harsher winter season, a serious shortage of plants may arise in the spring. If the stock has to be resown in spring, this can already represent a significant additional cost. Whether these increased costs are offset by the higher and more stable yields of autumn-sown crops depends primarily on the region and year, but farmers will only choose this path if they have a sufficient income from cultivation. For example, studies demonstrate that specific cultivars, like winter safflower, can yield significantly better under suitable conditions, reflecting economic advantages despite the higher initial seed costs. However, yields can suffer dramatically if crops do not survive winter conditions, emphasizing the need for farmers to carefully assess and manage the risks involved in autumn sowing, particularly given climatic variability [54,55].

In a broader context, pricing analysis indicates that the economic viability of autumn sowing is also influenced by market trends for winter-seeded crops compared to spring options. Increased competition for resources during planting seasons and fluctuating demand for winter crops can significantly impact profitability [56]. Furthermore, ensuring proper nitrogen management is critical, as poorly managed nitrogen can further diminish the resilience of winter crops and thus their economic viability [57]. Although growing varieties with adequate winter hardiness can significantly reduce the risk of loss of income, biotic stress factors occurring in early spring can pose additional threats.

## 3. Biotic Stress Factors Affecting Overwintering Plants

### 3.1. Diseases That Threaten Overwintering Plants

Winter damage to plants, such as frost cracks on stems, can significantly increase their vulnerability to diseases and pests in the early spring. This vulnerability is particularly evident when these wounds serve as entry points for fungal pathogens. When plants experience winter injuries, such as frost cracks or mechanical damage, their defensive capabilities are often compromised. This condition can make them more susceptible to infections from various fungal species, which can enter the injured tissues. These pathogens can colonize the vascular tissue through hyphal growth or sporulation, particularly under favorable environmental conditions, such as the cool, damp weather often experienced in spring [58]. The wounding activates stress responses that may ultimately weaken the plant’s defenses against subsequent infections [59].

Environmental factors, like extended periods of cold or fluctuations in temperature, further exacerbate this situation. In plants exposed to osmotic stress, signaling pathways can be rapidly activated [60]. Such stress factors can weaken the plants’ response to infecting pathogens, as they divert their resources toward repairing damage instead of developing an effective defensive reaction. Stress responses in plants are closely linked to injury and disease susceptibility, noting that disturbances in physiological functions can compound vulnerabilities in stressed plants [61].

The dynamics of fungal infections are illustrated by findings regarding *Phoma sclerotioides*, which were found to correlate with alfalfa plants suffering from winter injury [62]. Although some studies suggest that the fungus may take time to develop symptoms in affected plants, others indicate that disease can manifest quickly under conducive conditions, underscoring how winter-related damage weakens plant structure and facilitates a hospitable environment for diseases. Additionally, *Schizophyllum commune*, a wood-decomposing fungus, thrives in environments created by winter injuries, accessing living plant tissues through frost-induced cracks [63]. In practical terms, this means that plants weakened by winter damage might show symptoms of disease almost immediately upon the arrival of favorable weather conditions—sometimes within one season, stress can lead to rapid pathogen proliferation [64]. But effective management of these winter injuries through good horticultural practices may help mitigate the risk of fungal infections in early spring.

### 3.2. Pests Dangerous to Overwintering Plants

In spring, physiologically stressed plants that have overwintered are particularly vulnerable to pests such as aphids and vine weevils (*Otiorhynchus* spp.), as the combination of reduced plant vigor and environmental conditions conducive to pest development facilitates rapid pest population increase. Weakened plants, which may have experienced damage from previous seasons or stressors like drought, typically show reduced resistance, making them more susceptible to pest infestations. Root weevils such as *Diaprepes abbreviatus* can inflict significant damage through larval feeding, undermining the plant’s root systems and leading to a higher incidence of diseases such as *Phytophthora root rot* [65,66]. Root weevils and other pests like *Sitona lepidus* exploit weakened plants, allowing for higher reproductive rates and increased pest populations [67]. Aphids, which are phloem feeders, are also likely to proliferate on stressed plants. Elevated CO_2_ levels and nitrogen availability significantly influence aphid dynamics. Increased CO_2_ can enhance plant growth and biomass, but it can also lead to changes in phloem composition that may either favor or inhibit aphid reproduction depending on other environmental conditions [68,69,70]. Studies indicate that nitrogen content in plants can drive aphid abundance, suggesting that weakened plants may accumulate higher concentrations of nutrients under unfavorable conditions, making them more appealing to pests [69,70]. Furthermore, research has shown that stressors such as drought can make plants more suitable as food sources for herbivorous insects, thereby accentuating risks for weakened populations [71].

Climate change also plays a pivotal role in altering plant–pest dynamics. Warmer temperatures can increase aphid population growth rates while affecting their metabolic processes and survivability [72,73]. Spring allows for a rapid increase in pest populations, as milder temperatures can lead to quicker developmental rates for pests, further threatening already vulnerable plants. Thus, the biotic stress from pest populations, amplified by abiotic stressors like water scarcity or temperature extremes, positions overwintered plants at higher risk during the spring season [73].

## 4. Mechanisms Playing a Role in Winter Hardiness of Annual, Herbaceous Plants Sown in Autumn

Although frequently used in the literature, the term ‘winter hardiness’ is difficult to define precisely. The ability of plants to survive winter periods depends not only on the lowest temperatures, including frost tolerance (the ability to survive temperatures below the freezing point), but also on the interplay of several other factors. These comprise the length of the acclimation period, humidity, periods of prevailing warm temperatures, frost factors, and stress factors, including damage by pathogens and pests [35].

Winter hardiness is therefore a very complex trait and is often confused with frost tolerance. The complexity of winter hardiness in autumn-sown crops is due to the fact that they have to tolerate a number of adverse environmental factors, such as the presence/absence of snow cover, soil movement (the lifting of the soil due to freezing), or adverse conditions caused by ice cover. Plants weakened in winter must also have good resistance to biotic factors, such as certain diseases and pests [74]. However, one of the most important traits is indeed frost tolerance (tolerance of temperatures below freezing), which largely determines the survival of plants in winter.

### 4.1. Mechanisms Involved in Frost Tolerance

Frost tolerance is the ability of a plant to tolerate below-freezing temperatures, including the formation of ice crystals, without direct damage. One of the most serious consequences of frost damage is the formation of ice crystals in the extracellular space, which can lead to damage to the cell membrane and dehydration due to the outflow of water from the cell [75,76].

The mechanism of frost tolerance involves mainly short-term physiological responses. Many plant species are able to adapt to temperatures below freezing, especially temperate and cold climate plants. In the case of these plants, frost resistance can be significantly increased by certain environmental stimuli, e.g., low temperature and short-day illumination [75]. In annual herbaceous plants, frost tolerance is typically acquired by autumn cold induction and involves a number of adaptive mechanisms [76]. The process of adaptation mechanism is called cold acclimation or frost hardening [75].

During freezing, the ice formation frequently is initiated in the extracellular space, resulting in water leakage from the cells due to decreasing water potential, which leads to cell dehydration. Due to the decrease in the water content of the cells, the same processes occur as observed during desiccation stress: the cell volume decreases, the cytoplasm becomes denser, and macromolecules become unstable. Therefore, frost tolerance actually requires the plant to tolerate desiccation. The desiccation caused by frost creates a situation that plants can protect themselves from with their mechanisms based on desiccation tolerance [77]. The physiological and molecular responses of frost tolerance and drought tolerance stress tolerance show a high degree of overlap, and drought tolerance mechanisms are believed to form the basis of frost tolerance mechanisms. This is also indicated by the fact that many genes that respond to cold stress respond to drought and osmotic stresses as well [78].

### 4.2. Mechanisms Involved in Winter Hardiness

The term ‘winter hardiness’ means the long-term survival of the entire plant system during the winter months. In the case of annual herbaceous crops, it includes resistance to frost, long-term snow cover (lack of fresh air), desiccation caused by wind and solar radiation occurring below frost temperature, soil movement due to freeze–thaw cycles, and pathogens, which attack the weak plants in winter and early spring [79]. A broad range of ecological, anatomical, and physiological factors play a role in the mechanism for adaptation to the vicissitudes of winter.

Among them, the most important is the development of the aforementioned frost tolerance, which involves the production and accumulation of various metabolites, as mentioned in the previous section. The accumulation of carbohydrates can also function as a reserve nutrient for the start of spring growth/development. The accumulated carbohydrates may also play a role in snow cover tolerance [79]. Several other factors have a significant effect on tolerance to snow cover, such as growth habit and morphological characteristics. For example, in the case of faba beans, the varieties with compact growth habits and stems with short internodal parts and petioles and small leaves tolerate snow cover better than other types. Varieties with this phenotype are also more tolerant of windy winter weather [79]. Low relative water content and slow growth traits were also linked to better winter hardiness [79].

Summarizing, by virtue of this wide range of stressful conditions that a plant may experience during the cold season, winter hardiness is a complex trait [80]. Freezing temperature is the most relevant stress factor, although other stress situations, such as anoxia due to excess water or to ice encasement and photoinhibition due to the combination of light and low temperature, may also occur [80]. The adaptation to cold climate can be achieved either by the development of a powerful frost tolerance ability or by limiting the life cycle to the short summer season [80]. However, to improve winter hardiness, it is essential to understand the physiological changes that occur under the influence of low temperatures.

## 5. Changes Induced by Low Temperature

### 5.1. Cell Wall Structure Changes Under Low-Temperature Stress

Low-temperature stress has a significant impact on plant metabolism and development, particularly at the cellular level, where it induces extensive remodeling of the cell wall. As the primary structural and defensive barrier against abiotic stress, the cell wall undergoes dynamic and tightly regulated changes upon cold exposure. These changes play a central role in enhancing cold tolerance, as they contribute to increased mechanical stability, reduced cell damage, and improved overall resilience during freeze–thaw cycles.

Cold adaptation is often associated with increased deposition of key structural components within the cell wall. Early work showed that extensin mRNA expression is upregulated at low temperatures, leading to the accumulation of extensins and other cell wall-associated compounds such as callose, cellulose, and lignin. This increased deposition contributes to the mechanical reinforcement of the cell wall, thereby improving its resistance to mechanical stresses and dehydration associated with freezing [81]. Among these components, callose—a β-1,3-glucan—plays a particularly important role as a rapidly inducible, stress-responsive polymer. Cold exposure has been shown to trigger significant callose deposition in *Arabidopsis thaliana*, reinforcing cell wall structure and enhancing defense capacity, especially against opportunistic pathogens that frequently exploit tissues weakened by abiotic stress [82].

Low-temperature stress also influences the biosynthesis of cellulose, a major determinant of cell wall strength. Overexpression studies have revealed that cold conditions activate genes associated with cellulose production; for instance, MaNAC1 overexpression in banana promotes the transcription of cellulose biosynthesis genes, leading to an increase in cellulosic glucan content during chilling stress [83]. Such increases in cellulose deposition ensure structural integrity at a time when cell walls are subject to substantial tensile forces generated by ice formation and cellular dehydration.

Lignin accumulation represents another hallmark of cold-induced cell wall remodeling. As a critical structural polymer, lignin enhances rigidity and reduces wall permeability, contributing to the mitigation of freeze-related mechanical stress and protecting tissues from ice-induced dehydration. Studies have consistently documented increased lignification under cold exposure, reinforcing the dual role of lignin in mediating both abiotic stress tolerance and resistance to cold-associated pathogen pressures [84].

The remodeling of the cell wall under cold stress is further facilitated by the differential regulation of cell wall–modifying enzymes. Enzymes such as peroxidases and pectin methylesterases contribute to increased cross-linking among wall polysaccharides and phenolic compounds, ultimately strengthening wall structure and enhancing resistance to ice-induced mechanical strain [85]. This enzymatic reconfiguration is essential for maintaining cell wall plasticity while ensuring adequate structural reinforcement.

### 5.2. Accumulation of Osmoprotectants

Adaptation mechanisms related to frost tolerance involve osmoregulation, which means accumulation of soluble sugars and amino acids, e.g., proline [86]. Since most abiotic stresses can induce significant changes in the osmotic pressure of cells, osmoprotectant molecules play a prominent role in the development of tolerance [87]. Osmoprotectants actually maintain osmotic balance and help with freezing stress tolerance, all without interfering with enzyme activity or causing any toxic effects [87]. The prevention of frost damage is also supported by special so-called antifreeze proteins, which also play an important role in the frost adaptation mechanism. These kinds of proteins are found in fish, insects and plants, and their effect is based on the prevention of damage to cells caused by ice crystals [88]. The plant antifreeze proteins can bind to ice crystals and effectively prevent their growth and inhibit recrystallization [88].

### 5.3. Changes in Membrane Lipids

Dehydration due to freezing can also cause severe membrane damage [89]. The membrane lipid composition can show remarkable changes in response to subfreezing temperatures, and this process contributes significantly to frost stress adaptation and helps plants survive.

Low-temperature stress induces pronounced alterations in polyunsaturated fatty acid (PUFA) metabolism across plants and other cold-exposed organisms, reflecting a central adaptive mechanism for maintaining membrane functionality. A key response involves the upregulation of fatty acid desaturases, which increase the degree of lipid unsaturation and thereby preserve membrane fluidity under cold conditions. For instance, enhanced expression of ω-3 fatty acid desaturase genes such as GmFAD3A has been shown to promote PUFA accumulation and improve cold tolerance in rice [90]. Similar adjustments in membrane lipid composition have been documented in microalgae, where cold stress stimulates PUFA biosynthesis via the Kennedy pathway, ensuring the maintenance of membrane integrity during temperature decline [91].

Cold-tolerant cultivars also exhibit characteristic changes in lipid unsaturation. Winter-hardy Bermuda grass, for example, displays increased PUFA levels during cold acclimation, a modification associated with enhanced membrane stability and improved overwintering capacity [92]. Environmental conditions further modulate these responses; cold exposure redirects fatty acid metabolism toward unsaturated lipid production, thereby reinforcing membrane flexibility and cellular resilience [93]. For example, in barley plants, major increases in the levels of phosphatidic acid (PA), lysophosphatidic acid (LPA), and monogalactosyl diacylglycerol (MGDG) have been shown in frost-tolerant varieties [94].

These physiological and biochemical shifts are not restricted to plants. Early research established that cold acclimation in fish is accompanied by increased membrane lipid unsaturation, a response consistent with the broader biological strategy of maintaining functional membrane dynamics under cold stress [95]. Comparable mechanisms have been identified in psychrophilic microorganisms: genomic analyses of *Glaciozyma antarctica* highlight the role of desaturase-mediated PUFA synthesis in sustaining membrane fluidity in persistently cold environments [96].

## 6. Autumn-Sown Legume Crops Compared to Spring-Sown Plants

*“Beans, as was said, are in other ways not a burdensome crop to the ground, they even seem to manure it, because the plant is of loose growth and rots easily”*

(Theophrastus, 3rd century BC)

### 6.1. The Economic and Ecological Benefits of Integrating Legumes into Cropping Systems

In recent agronomic research, comparisons between winter-hardy legumes and traditional non-leguminous crops such as wheat and rapeseed highlight the substantial contributions that involve leguminous crops in crop rotation, playing an important role in the development of biodiversity-based agriculture (BBA) [97].

These contributions include improvements in soil health, nitrogen availability, and subsequent crop performance, positioning legumes as valuable—though context-dependent—components of sustainable agricultural practices.

Legumes possess the unique ability to fix atmospheric nitrogen through symbiotic associations with rhizobia, thereby enhancing soil nitrogen availability for subsequent crops such as winter wheat. Leguminous plants as previous crops, including soybean, can increase both the yield and gluten protein content of winter wheat while concurrently improving soil properties such as soil organic matter (SOM) and pH [98]. Similar findings indicate that legume-based rotations can maintain yield advantages across diverse cropping sequences [99]. These outcomes are particularly relevant given the stagnation in wheat yield trends observed in regions where legumes have been progressively left out of crop rotations.

The integration of winter-hardy legumes—such as winter pea—into rotational systems also influences soil microbial communities and enzyme activities. Winter pea, compared to rapeseed, enhanced soil fertility indicators and supported improved nitrogen balance, ultimately contributing to higher subsequent wheat yields [100]. The capacity of legumes to conserve soil nitrogen is reflected in the findings of [101], who documented higher post-harvest soil nitrate levels following legume crops relative to non-leguminous alternatives, underscoring their superior role in nutrient cycling and soil productivity. It is important to note, however, that rapid mineralization of legume residues may increase the risk of nitrogen losses under certain environmental conditions, suggesting that management practices must be carefully optimized.

In contrast, traditional crops such as rapeseed and wheat present challenges in terms of nitrogen management and soil health. Studies showed that catch crops following winter oilseed rape can reduce nitrate leaching, although the degree of nitrogen retention achieved with legume-based cover crops was generally greater [102]. Such comparisons illustrate how legumes can mitigate some environmental drawbacks associated with non-leguminous cropping systems, while also highlighting the need to consider site-specific responses and crop management strategies.

Legumes may additionally contribute to improved water use efficiency within rotational systems. The inclusion of crops with contrasting root architectures—including legumes—can increase water use efficiency by 10–25%, an important consideration in drought-prone agroecosystems [103]. While these effects are not exclusive to legumes, their typically deep or branching root systems can complement other crops in rotation.

Finally, studies have underscored the economic and ecological benefits of integrating legumes into cropping systems. Reductions in herbicide dependency and increases in biodiversity are associated with legume-based systems, suggesting that legumes contribute not only to yield stability but also to environmentally sound management practices [104]. Intercropping evidence further supports these findings; pairing legumes with cereals such as wheat has been shown to enhance both forage yield and quality [105,106]. However, the successful implementation of legumes in rotation depends on factors such as cultivar winter hardiness, disease pressure, and climatic suitability, which may limit performance in certain environments.

In summary, legumes play a key role in sustainable agriculture due to their nitrogen fixation, their ability to improve soil structure and water management, and their contribution to protein-rich food and feed systems. In temperate and Mediterranean regions, the sowing season—autumn or spring—influences crop performance, phenology, yield potential, and resilience to environmental stress. In recent decades, increasing climate variability and pressure for more sustainable systems have renewed interest in autumn-sown legumes, which in many cases have shown better agronomic and ecological performance compared to their spring-sown counterparts [19,107].

The capacity of leguminous cover crops to cycle carbon, nitrogen, and phosphorus positions them as a critical element in sustainable agricultural systems, contributing to increased soil productivity [108]. The environmental footprint of animal protein production is substantial, driving greenhouse gas emissions, excessive water consumption, and deforestation. Although legumes provide a high-protein, sustainable alternative, they remain a small component of most diets in developed or high-income countries. With their cultural significance, minimal environmental impact (low greenhouse gas and water footprints), soil-enriching nitrogen fixation, and affordability, legumes are a valuable and sustainable protein option [109]. Enhancing nutrition is a key development priority, especially in low- and middle-income countries across Africa and South Asia, where undernutrition remains a challenge alongside rising obesity rates. Legumes can play a crucial role in the transformation of food systems and enhance nutrition in these regions. Given their numerous benefits, pulses should be more prominently integrated into future cropping and food systems [110]. Integrating legumes in rotations in cereal-based cropping systems increases the yield and quality of cereals. Similarly, integrating leguminous species in pastures increases the yield and quality of grass pastures [111].

#### 6.1.1. Effect of Sowing Time on Legume Crops

In the case of legumes, several species can also be sown in both autumn and spring, and the frost tolerance limit is usually about −8 and −9 °C. Lentils (*Lens culinaris Medik.* subsp. *culinaris*) and faba beans (*Vicia faba* L.) are the most sensitive; peas tolerate the cold better, whereas chickpeas (*Cicer arietinum* L.) have the best winter hardiness [112]. In the Pannonian region, for example, among legumes, autumn sowing of both peas (*Pisum sativum* L.) and faba beans (*Faba bean* L.) is recommended, partly due to their high winter survival rate and partly due to other beneficial effects, such as longer soil covering, early flowering, and higher yields compared to spring sowing [17]. Autumn sowing significantly increases yield, biomass, and nitrogen fixation in cool-season grain legumes compared to spring sowing (Table 3).

However, the survival rate was strongly dependent on the genotype and its cold tolerance and crop year [13]. Moreover, considering the winter survival rate of pea, faba bean, and white lupine genotypes, much greater differences were detectable within species than between species [51]. The response of the genotypes to the sowing date can be significantly different even if the cultivation takes place on the border of the temperate climate zone, e.g., in the case of soybeans (*Glycine max* L. Merril) in subtropical conditions (Brazil) [113]. In addition, significant differences could be detected between winter pea genotypes in branching capacity, plant height, number of pods and seeds, seed and biological yield, etc. [114].

#### 6.1.2. Effect of Sowing Time on Yield

The dry matter yields of aboveground biomass and grain yield were significantly higher than those of spring-sown winter faba bean, winter pea [13], and chickpea [115]. Some winter-type faba beans have shown higher grain yield per pod and/or higher grain yield per shoot. In the Mediterranean region, winter-sown lentils and chickpeas produced 50–70% more seed yield than the spring-sown crop [115,116]. Peas sown in autumn may produce significantly more (even by 56%) yield compared to those sown in spring [117]. The longer growing period of the winter-sown crop resulted in higher biomass production, which contributed mainly toward increased seed yield [115]. In forage pea, the genotype, crop year, and sowing time (spring or autumn) influenced the forage yield and dry matter content [118]. Both fresh forage yield and dry matter content of forage pea were significantly higher when cultivars were sown in autumn compared to those sown in spring [118]. In general, the longer growing period of winter-sown legume crops resulted in higher biomass production, which contributed mainly toward increased seed yield [115]. However, differences in yield and yield-related parameters were highly varied depending on genotype and crop year [118]. Although the winter cold and frost did not damage any winter pea breeding line in either year, the crop year had a significant effect on yield and yield-related traits. The differences among years were primarily caused by differences in temperature and precipitation of crop years that occurred during the active growth period in the spring [114].

#### 6.1.3. Effect of Sowing Time on Developmental and Nitrogen Fixation Characteristics

Temperate cool-season legumes such as peas, faba beans, lupines (*Lupinus* spp.), and chickpeas are long-day plants in terms of flowering and may be sensitive to vernalization [119,120,121,122,123,124]. The alleles responsible for these traits can influence the phenological and agronomic properties of crops under autumn and spring sowing conditions [125]. Plants of winter faba bean and winter pea were, in general, at the 5–7 leafy stage at the time of sowing the spring cultivar [17]. During the flowering period, the differences in their developmental stage reduced, and winter types ripened about 1–2 weeks earlier than spring types [17]. In contrast, the ‘Hiverna’ cultivar of faba bean did not differ in the date of maturity compared to the spring-type cultivar Alexia, although its development preceded it until the end of the flowering period [13]. Significant variability could be observed in growth vigor during spring between pea genotypes [126]. Both the duration of the period till flowering and the ripening of winter pea were influenced by genotype and location of the experiment inside the Danube region in Bulgaria, resulting in 16–17 days’ differences in vegetation period [127].

Autumn-sown faba bean varieties ‘Diva’ and ‘Hiverna’ had more biologically bound nitrogen remaining in the soil and were less sensitive to seeding rate compared to spring-sown faba bean variety ‘Alexia’ [128]. Similarly, the nitrogen fixation and nitrogen yield of autumn varieties (‘Aviron’, ‘Cherokee’, ‘Curling’, ‘Enduro’, ‘Isard’, ‘James’) were much higher than that of the spring type (‘Astronaute’) in peas [129].

**Table 3:** Effects of sowing time on cool-season grain legumes—key parameters.

Crop	Key Benefits of Autumn Sowing	Location	Reference
Faba bean	Much higher biomass and grain yield; More seeds per pod and per shoot; More N left in soil after harvest; Ripens 1–2 weeks earlier.	Pannonian region, Austria	[13,17,128]
Pea	Up to 56% higher grain yield; Higher fresh forage and dry matter; Stronger N-fixation and N-yield; More branches, taller plants, more pods/seeds	Greece, Turkey, Pannonian	[114,117,118,129]
Lentil	50–70% higher seed yield	Mediterranean	[115,116]
Chickpea	50–70% higher seed yield; Higher biomass	Mediterranean	[115]
White lupine	Winter survival varies more within varieties than between species	Temperate	[51]
All legumes	Longer growing season; More biomass and yield; Deeper roots, better water use; Chickpea most frost-tolerant; Lentil and faba bean most sensitive	Temperate/Mediterranean	[17,112]

## 7. Breeding of Legumes for Winter Hardiness

In this review, we aimed to collect and process the literature on breeding legume crops for overwintering. Due to global warming, there is a tendency to change the sowing time of some spring-sown legumes to autumn sowing in the temperate zone [115,130]. Although autumn sowing can have several advantages, it requires the development of new cultivars characterized by reliable overwintering ability for widespread cultivation of autumn-sown legumes [131]. One of the most significant limiting factors for growing autumn-sown crops in the temperate zone is the winter hardiness of the varieties [132]. Breeding for winter hardiness requires reliable evaluation methods of traits related to winter hardiness.

### 7.1. Assessing Winter Hardiness

Winter hardiness in grain legumes is assessed using a combination of field assessments and controlled laboratory/artificial freezing tests. Key metrics include visual scoring of damage, such as leaf chlorosis and regrowth potential after a cold event, and physiological indicators like electrolyte leakage from cell membranes and chlorophyll fluorescence. Lethal Temperature 50 (LT50), the temperature at which 50% of plants die, is a common quantitative measure derived from controlled freezing experiments. Other factors like proline content, sugar concentration, and changes in fatty acids can also provide insights into cold tolerance.

Under natural conditions, field-based evaluations offer direct insights into plant performance following winter exposure. Visual scoring is one of the most widely used approaches, involving the observation of frost damage symptoms such as yellowing (chlorosis), reduced height, or lack of regrowth after winter [133]. Another critical measure is survival rate, which is the percentage of plants that survive the winter under natural conditions and provides a direct measure of hardiness [134]. Furthermore, comparative field trials enable the establishment of performance rankings among genotypes, allowing breeders to identify varieties with superior winter hardiness [112].

In addition to field observations, laboratory or artificial freezing experiments allow for the quantification of cold tolerance under controlled conditions. Plants or excised tissues are exposed to progressively lower temperatures to determine the LT50—the temperature at which 50% of the tested samples fail to survive [112,135]. This parameter serves as a key quantitative indicator of frost tolerance.

Physiological assays further complement freezing tests. Electrolyte leakage analysis measures the release of electrolytes from damaged cell membranes, with higher leakage indicating greater cold injury [136]. Similarly, chlorophyll fluorescence measurements are widely used to assess photosynthetic performance under freezing conditions, providing insights into the functional stability of photosystem II [28,136].

### 7.2. Monitoring Biochemical Changes

Assessing cold stress in plants requires an integrative approach that captures physiological, biochemical, and molecular responses to low temperatures. Physiological indicators such as Differential Thermal Analysis (DTA) are widely used to determine the freezing point of plant tissues and quantify cold hardiness, enabling the evaluation of genotype- and environment-dependent variation in stress responses [137]. Measurements of cell turgor and water relations further reflect the mechanical and osmotic adjustments associated with cold acclimation, as increased cell tension has been correlated with enhanced freezing resistance [138].

Cold acclimation in grain legumes is often accompanied by characteristic biochemical adjustments. The accumulation of proline, an osmoprotectant amino acid, is a well-documented physiological response associated with enhanced frost resistance [135,136]. Moreover, changes in sugar concentration (positively correlated with tolerance in pea) and the composition of membrane-bound polyunsaturated fatty acids can indicate cold acclimation [112,136]. Winter-hardy legumes produce high levels of osmoprotectants like sugars (glucose, sucrose, raffinose, fructose) and suberin. These compounds help maintain cellular osmotic balance and prevent cold-induced cellular damage by protecting cellular structures from freezing and dehydration [28].

Biochemical characterizations provide insight into cold-induced modifications of the cell wall. Quantification of polysaccharides such as pectin and cellulose reveals structural adjustments linked to improved cold tolerance [139]. Enzyme assays targeting polygalacturonase and pectin methylesterase capture changes in pectin metabolism, which are essential for maintaining wall integrity under chilling stress [140].

At the molecular level, transcriptomic profiling enables the identification of cold-responsive pathways, including the induction of CBF- and GST-related genes that underpin acclimation processes [141]. Complementary proteomic analyses further document cold-induced alterations in extracellular and cell wall-associated proteins, highlighting molecular remodeling events that contribute to enhanced resilience [142]. Structural analyses of the cell wall offer direct evidence of cold-induced remodeling. Techniques such as scanning electron microscopy reveal changes in wall rigidity and surface morphology under freezing conditions [143], while chromatographic and spectrometric analyses provide detailed information on shifts in lignin, hemicellulose, and other matrix components [144].

Finally, integrative indices such as the Chilling Sensitivity Index (CSI) synthesize physiological parameters, including growth performance, to quantify genotypic variation in cold sensitivity and support comparative assessments across plant species and cultivars.

### 7.3. Determining Antioxidant Activity

The determination of antioxidant activity in plant materials, including faba bean, typically relies on a suite of complementary analytical approaches that capture different aspects of antioxidant function. Among these, the DPPH (2,2-diphenyl-1-picrylhydrazyl) radical scavenging assay remains one of the most widely applied methods for evaluating free radical quenching capacity. In this assay, the reduction in absorbance at approximately 517 nm, caused by the interaction between DPPH and antioxidant-containing extracts, provides a rapid and effective indication of radical scavenging potential [145]. A similar principle underlies the ABTS (2,2′-azino-bis(3-ethylbenzothiazoline-6-sulphonic acid)) assay, in which the decrease in absorbance at 734 nm following exposure of the ABTS•^+^ radical cation to plant extracts is quantified to assess antioxidant activity. Owing to its applicability in both hydrophilic and lipophilic systems, the ABTS assay is frequently employed in studies seeking biologically relevant estimations of antioxidant capacity.

In addition to radical scavenging methods, assays based on reducing power offer further insight into antioxidant mechanisms. The FRAP (Ferric Reducing Antioxidant Power) assay quantifies the ability of compounds to reduce Fe^3+^ to Fe^2+^, with increased absorbance at 593 nm serving as an indicator of higher reducing capacity [146]. The ORAC (Oxygen Radical Absorbance Capacity) assay provides a more physiologically oriented assessment by measuring the capacity of antioxidants to neutralize peroxyl radicals, using fluorescence decay as a proxy for antioxidant efficacy.

Beyond chemical assays, cell-based methods have gained prominence for their ability to account for bioavailability, cellular uptake, and metabolic interactions. Such approaches typically involve exposing cultured cells to oxidative stress—often induced by hydrogen peroxide—and evaluating the protective effects of extracts through viability indicators such as MTT reduction or LDH leakage. These assays offer greater biological relevance and may better reflect the functional antioxidant potential of plant-derived compounds in vivo.

Although not direct measures of antioxidant activity, the quantification of total phenolic and flavonoid content using the Folin–Ciocalteu reagent and aluminum chloride colorimetry, respectively, remains an important complementary approach. The abundance of these compounds is frequently correlated with antioxidant capacity, thereby providing a useful proxy for assessing the potential bioactivity of plant materials [146]. Additionally, in legume crops such as faba bean, measurements of chlorophyll content and its modulation under stress conditions have been used to explore the relationship between pigment degradation, oxidative stress, and antioxidant responses. Such analyses contribute to a more integrated understanding of plant resilience under biotic and abiotic stressors [147,148].

### 7.4. Factors Influencing Winter Hardiness

Winter hardiness is influenced by genetic, physiological, and environmental factors, such as crop species, crop genotype, crop acclimatization, plant structure, protective snow cover, and winter temperatures [17,28,133,135,149]. Winter hardiness in cool-season grain legumes is influenced by numerous genetic, physiological, and environmental factors (Table 4).

#### 7.4.1. Crop Species

Among grain legumes, faba bean and lentil showed the highest winter hardiness, followed by pea and chickpea [112]. Among peas and white lupine, pea plants typically display higher frost tolerance than white lupine, with peas having a mean LT50 (lethal temperature for 50% mortality) of −12.8 °C compared to −11.0 °C for white lupine [150]. It was found that selected wild *Cicer* species had more freezing tolerance than well-known cold-tolerant chickpea cultivars [151]. The importance of developing winter-hardy varieties of faba bean (*Vicia faba*) becomes increasingly evident when considering their potential contribution to yield stability and crop resilience in agriculture. As a legume that is commonly sown in autumn, faba beans can provide significant benefits when bred for winter hardiness, particularly in the context of improved agronomic traits and climate adaptability.

One critical aspect of winter hardiness is the ability of faba bean to endure cold temperatures, which is vital for survival and productivity after overwintering. Frost tolerance is an essential component of winter hardiness in faba beans, with sufficient seedling development and cold acclimation being critical before hard frosts [152]. This is particularly relevant in northern climates where winter conditions can be harsh. Additionally, the genomic studies identified quantitative trait loci (QTL) related to freezing tolerance in winter faba bean breeding, indicating the potential for significant genetic gains in winter hardiness through selective breeding [153].

Moreover, the assessment of agronomic performance and adaptability under varying environmental conditions shows that winter-hardy faba beans can better cope with stressors, thereby positively influencing overall crop yield. This adaptability is crucial, as climate variability poses challenges to traditional cropping systems, making winter-hardy cultivars increasingly attractive to farmers aiming for more reliable yields [154].

Faba bean yield and quality indicate that winter-hardy varieties can also yield high protein contents, suggesting that both the quantity and quality of the crop can benefit from winter hardiness [155]. Winter-hardy faba beans outperform traditional spring-sown varieties, particularly in terms of yield consistency across fluctuating temperatures. Winter types contribute to better protein and grain yields, underscoring the importance of breeding winter-hardy lines for improved food security [156]. Furthermore, cultivar trials have demonstrated that well-adapted faba bean cultivars can thrive in various climatic conditions, enhancing their potential for both urban and rural agricultural practices [23].

#### 7.4.2. Genotype Variation

Within pea and lupine, there are significant genetic differences in frost tolerance, with genotype LT50 values for peas ranging from −11.6 °C to −14.5 °C [150]. Screening of 109 accessions of faba bean, three accessions of narbon bean (*Vicia narbonensis* L.), and two accessions of *Vicia montbretii* Fisch. et C.A. Mey. showed a considerable variation for cold tolerance in faba beans, and wild relatives of faba bean were found to be more tolerant to cold than those of cultivated faba beans [157]. Several Australian-bred varieties and elite lines have significantly better frost tolerance than the local Saudi variety [158]. 11 genotypes of pea for winter hardiness showed the significant difference among genotypes for LT50 value, plant mortality proportion at the two lowest freezing temperatures, and biomass injury visual score (VS) after the highest and lowest freezing temperatures and classified the 11 pea genotypes into three winter hardiness classes based on field-based winter mortality data. The pea genotypes KI_L38, Isard, Champagne, Dolmen, and KA_37 were identified with a high level of winter hardiness [150].

#### 7.4.3. Acclimatization

Acclimatization improves winter hardiness in grain legumes by triggering physiological and molecular changes that increase freezing tolerance. This process involves plants adapting to low non-freezing temperatures by reducing shoot growth, decreasing tissue water content, altering membrane lipid composition, accumulating protectant compounds like sugars and proline, activating cold-responsive genes (like the ICE-CBF-COR pathway), and enhancing their antioxidant system [136,159,160,161]. These adaptations allow winter-type legumes to sustain metabolic activity and avoid cellular damage when temperatures drop below freezing. This process allows plants to adapt to gradually decreasing temperatures before the onset of winter, increasing their survival potential. However, variable winter temperatures can induce cycles of de-acclimation and reacclimation, which can negatively affect survival by repeatedly exposing plants to stress or causing premature loss of tolerance [160,161].

The ICE-CBF-COR signaling pathway is a key regulatory cascade in plants that responds to cold stress, enabling them to acclimatize and survive freezing temperatures. This pathway includes the Inducer of CBF Expression 1 (ICE1), which acts as a master regulator, leading to the expression of cold-responsive genes, specifically the C-repeat binding factor (CBF) genes, and subsequently cold-regulated (COR) genes that confer freezing tolerance. The ICE1 protein is a basic helix–loop–helix (bHLH) transcription factor that plays a crucial role in mediating plant responses to cold temperatures. Upon exposure to low temperatures, ICE1 is stabilized and can activate the expression of CBF genes, namely CBF1, CBF2, and CBF3 [162]. These genes are integral to the cold acclimation process, as they encode other transcription factors that regulate downstream COR genes [163]. The CBF proteins, upon activation, bind to specific cis-regulatory elements in the promoters of their target genes known as C-repeat/Dehydration-Responsive Elements (DREs) [164]. This binding facilitates transcriptional activation of various COR genes, which produce proteins that protect the plant cells from cold damage and assist in acclimatization [165]. For instance, CBF1 has been shown to induce the expression of multiple COR genes at non-acclimating temperatures, enhancing freezing tolerance in non-acclimated plants [166].

The ICE-CBF pathway does not function solely as a positive regulator of cold responses. Negative regulators such as HOS1 (Height of Leaf) target ICE1 for ubiquitination, leading to its degradation [163]. Additionally, MYB15 and other associated proteins can negatively regulate CBF expression under certain conditions [167]. Such regulation ensures that the response to cold stress is finely tuned, preventing unnecessary expenditure of resources when not required.

Under optimal conditions, JAZ (Jasmonate ZIM-domain) proteins repress ICE1 activity by physically interacting with it [168]. However, during cold stress, jasmonate signaling is triggered, leading to the degradation of JAZ proteins, which releases ICE1 to activate CBF genes [169]. This interaction illustrates the intricate cross-talk between various signaling pathways in plants, particularly in response to abiotic stresses.

Recent studies emphasize the interconnected nature of the ICE-CBF-COR pathway with other signaling cascades, such as those involving abscisic acid (ABA) and various kinases [170]. Organizational constellations within the pathway can also involve proteins like CAMTA3 that modulate the CBF response, indicating further complexity in the regulation of cold stress responses [167].

#### 7.4.4. Plant Structure and Phenology

Legume winter hardiness is influenced by a plant’s structure, such as root and shoot systems and phenology (timing of life cycle events like leafing out and flowering), which allows for cold acclimation [171,172]. Well-developed crowns, which contain the plant’s growing points, are essential for survival, with as few as 2–3 leaves being sufficient for survival if the crown remains protected below the freezing point of the soil. Winter pea lines can withstand winter conditions when they display rosette growth with small leaves and short internodes at the onset of winter [173]. Vernalization responsiveness, a trait determining when a plant flowers, can also enhance frost tolerance by preventing premature flowering in mild winters, as seen in some lupine species [171]. In the lupine breeding line ‘PLI-P3’, a high winter mortality was observed under field conditions; an early variety was associated with extreme earliness of flowering [174], a feature that would definitely increase its sensitivity to frost because of the early differentiation of the floral apex [175]. In pea, good intrinsic frost tolerance and extreme field-based winter survival [176,177] may be accounted for by frost escape under field conditions via delayed flowering.

#### 7.4.5. Snow Cover

**Figure 1:**
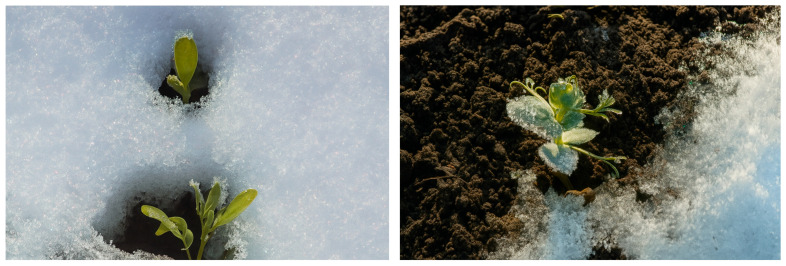
Winter legumes in the Research Institute of Nyíregyháza, University of Debrecen, Hungary (Source: pictures made by Nóra Mendler-Drienyovszki).

Snow cover (Figure 1) protects legumes from harsh winter conditions, acting as an insulating layer that prevents extreme cold and soil heaving, which can damage or kill plants. Soil heaving, caused by alternating freeze–thaw cycles, can disrupt root systems and is a major cause of winter kill in many crops, including some legumes. Snow cover reduces these freeze–thaw cycles, protecting the soil from disturbance [28].

#### 7.4.6. Winter Temperatures and Duration

Sustained freezing temperatures, especially at or below −6.7 °C, can cause significant yield losses in some grain legumes. Substantial seedling loss occurs for forage legumes below −6 °C and pinto beans below −3 °C. Even hardy species may not survive temperatures below −23 °C or −29 °C, depending on duration [133].

**Table 4:** Factors influencing winter hardiness in cool-season grain legumes.

Factor	Key Effect	Crop(s)	Reference
Crop species	Faba bean and lentil most hardy. Pea LT50 equals −12.8 °C. White lupin LT50 equals −11.0 °C. Wild *Cicer* more tolerant than cultivated chickpea.	Faba bean, lentil, pea, lupin, chickpea	[112,150,151]
Genotype variation	Pea LT50 ranges from −11.6 to −14.5 °C. Wild faba more tolerant than cultivated. Australian lines more tolerant than Saudi local. Pea divided into three hardiness classes.	Pea, faba bean, lupin	[150,157,158]
Acclimatization	Shoot growth decreases. Sugars and proline increase. ICE-CBF-COR pathway is activated. Antioxidant system increases. De-/reacclimation is risky.	General	[136,160,161]
Plant structure, phenology	Rosette growth helps winter pea. Early flowering increases mortality (lupine). Delayed flowering avoids frost.	Pea, lupine	[171,173,174]
Snow cover	Snow insulates plants. Snow prevents soil heaving.	General	[28]
Osmoprotectants	Sugars (glucose, sucrose, raffinose) increase. Suberin increases. These protect cells from freezing.	General	[28]
Winter temperature and duration	More than 50% loss below −6 °C (forage legumes). Hardy species survive −23 or −29 °C (depending on duration).	Forage legumes, pinto bean	[133]

## 8. Genetic Improvement of Winter Hardiness

Genetic improvements for winter hardiness in legumes are being pursued through traditional breeding, marker-assisted selection, and genomic techniques to identify genes controlling cold tolerance and utilize cold acclimation mechanisms. This research focuses on enhancing winter survival by selecting for traits like frost tolerance and cold acclimation potential, leveraging genetic diversity within species like winter pea, faba bean, and chickpea, and exploring complex genetic pathways to develop resilient cultivars for diverse environments and changing climates [28,134,160].

### 8.1. Traditional Breeding and Selection

Traditional breeding to improve cold tolerance in legumes involves crossing existing cold-tolerant and cold-sensitive lines, selecting for desirable traits from offspring, and utilizing landraces or wild germplasm as sources of novel cold tolerance genes. This process requires screening germplasm in either the field or controlled environments or both to identify and select plants with better survival under cold stress conditions, using phenotypic indicators, and then breeding the most promising line [28,134,178]. Key aspects of traditional breeding for cold tolerance involve germplasm screening, hybridization (crossing), and selection [116]. To identify sources of the resistance, germplasm consisting of a diverse collection of legume varieties, including landraces and wild relatives, was phenotyped for winter hardiness using visual scoring, chlorophyll fluorescence, and ion leakage assays in natural and/or controlled environments to assess cold stress responses. Crossing tolerant lines with well-adapted and sensitive lines to combine cold tolerance with desirable traits. Selection by visually assessing plants in the field or controlled settings to identify those that exhibit better survival, growth, and reduced damage under cold conditions [28,134,160].

Traditional breeding to improve cold tolerance in chickpea research has made great efforts to overcome the production constraints and to increase the productivity of chickpeas in Syria. This research program was a collaborative program between ICARDA and other national research organizations to improve the productivity of chickpeas. The first winter-sown chickpea variety developed by ICARDA and released in Syria in 1982 was Ghab 1 [179,180,181]. It was followed by a second variety, Ghab 2, in 1986; then Ghab 3 in 1991. Two other new varieties, Ghab 4 and Ghab 5, which have relatively larger seed sizes than Ghab 3, were released in 2002. The five new varieties offer the potential of considerably increasing national chickpea productivity. The new winter-sown varieties were developed to be resistant to both Ascochyta blight and cold. In over ten years of scientific trials, both on-station and on-farm, winter-sown chickpeas have consistently out-yielded the local spring-sown cultivars. The yield difference is usually between 50% and 100% [181,182]. The higher yields are due to a longer growing season, better utilization of moisture during growth and maturation, a higher germination rate, more favorable soil moisture and temperature conditions during reproductive growth, better nodulation, and less damage from insect pests [181].

Likewise, in lentils, in Turkey, traditional breeding and selection for the development of winter-hardy lentils were based on screening the existing germplasm. ‘Kışlık Pul 11’, ‘Kışlık Yeşil 21’, ‘Kışlık Kırmızı 51’, ‘Kafkas’, ‘Özbek’, ‘Çiftçi’, and ‘Kayı 91’ winter cultivars were selected from local landraces [183]. The winter-hardy lentil variety is ‘Morton’, a red lentil cultivar that can survive temperatures below −25 °F [184]. Other winter-hardy varieties developed and tested on-farm in Pakistan include ‘ILL 590’, ‘ILL 662’, ‘ILL 857’, ‘ILL 975’, and ‘ILL 1878’ [185], and the varieties ‘Hamria’ and ‘Bichette’ were released for moderate cold areas in Morocco [186]. These traditional breeding and selection results also indicate that the geographical origin of the genotypes could not directly be used to predict resilience to cold [187].

The currently good but not outstanding intrinsic frost tolerance of the pea landrace ‘Champagne’ in spite of its reportedly extreme field-based winter survival [176,177] may be accounted for by frost escape under field conditions via delayed flowering caused by possession of the *Hr* (high response to photoperiod) gene [188]. Indeed, the *Hr* gene reportedly co-segregated with the most important quantitative trait loci (QTL) for frost tolerance [189].

Even though the traditional breeding made tremendous progress in legume genetic improvement, cold tolerance is a complex trait influenced by multiple genes and pathways, making it challenging to improve through traditional methods alone. Traditional breeding needs to focus on traits like cold acclimation, de-acclimation, and reacclimation potential to survive seasonal temperature changes. Furthermore, understanding the genetic basis (e.g., through QTLs) helps to focus traditional breeding efforts [28,134,160,190].

### 8.2. Evaluation of Genotype–Environment Interactions and Polygenic Control

#### 8.2.1. Genotype–Environment Interactions Under Abiotic Stress

Effective breeding for improved tolerance to abiotic stresses such as cold, drought, and heat requires the integration of robust field evaluation, accurate characterization of genotype–environment interactions (GEI), and strategies capable of addressing the inherently polygenic nature of most stress-adaptive traits. Field-based phenotyping remains essential for capturing the true performance of genotypes under realistic environmental conditions. Multi-environment trials (MET) are particularly valuable in this context, as they enable the characterization of trait performance across diverse environments and thereby provide reliable estimates of stress responses. The importance of MET for dissecting complex traits, such as waterlogging tolerance in field pea [191]. Analytical tools such as GGE biplots further improve interpretation by visualizing the ‘which-won-where’ patterns of genotype performance [192]. Complementary controlled-environment assays can refine cold-tolerance phenotyping by reducing environmental variability; however, as emphasized, such tests may still be influenced by strong GEI and therefore should be interpreted alongside field data [193].

The complexity of GEI necessitates advanced statistical approaches. Graphical GEI models, including GGE biplots [194,195], facilitate the identification of broadly stable or specifically adapted genotypes. Meanwhile, additive main effects and multiplicative interaction (AMMI) models enable the partitioning of variability into genetic and environmental components, providing a more precise understanding of GEI patterns. The AMMI framework surpasses traditional analytical approaches in resolving GE interactions in multi-environment datasets [196].

#### 8.2.2. Genotype–Environment Interactions Under Biotic Stress

The relationship between biotic stress, manifested through damage by pathogens and pests, and abiotic stressors such as cold and freezing is a critical aspect of plant resilience and agricultural productivity. This interplay is significant because both types of stress affect plant health and immunity, often compounding the negative effects on growth and yield.

Biotic stresses arise from the intrusion of pathogens and herbivorous organisms that can inflict direct damage on plant tissues. Pathogenic microbes such as bacteria, fungi, and viruses exploit plant vulnerabilities, leading to significant agricultural losses globally [197]. Concurrently, abiotic stresses, particularly extreme temperatures, are substantial threats to plant survival. Cold stress can lead to physiological dysfunctions, including oxidative damage due to the accumulation of reactive oxygen species (ROS) [198]. For instance, in low-temperature environments, plants experience membrane instability, which exacerbates susceptibility to both abiotic and biotic stresses [199].

The interaction between these stressors can be detrimental; for instance, plants experiencing biotic stress often exhibit diminished physiological functions, reducing their ability to withstand abiotic stresses such as cold. This results in a feedback loop where biotic stress exacerbates the effects of abiotic stress and vice versa. Studies have demonstrated that the production of stress-related proteins, including antioxidants, helps mitigate the effects of both biotic and abiotic stressors [200,201]. These proteins are crucial as plants invest their energy in defense mechanisms, which could otherwise be utilized for growth and reproduction.

Moreover, the genetic and molecular responses of plants to these stress factors are intricately linked. Stress-responsive pathways are activated in response to cold but are influenced by prior exposure to biotic stress [202]. Proteins involved in signaling pathways related to abiotic stress often intersect with those involved in biotic stress responses, leading to coordinated regulation of plant defense mechanisms [203]. For instance, plants that endure biotic assaults may not only exhibit immediate defensive responses but also alter their physiological and metabolic states to better cope with cold stress.

Additionally, the expression of genes related to cold tolerance may be downregulated when plants are under attack from biotic stressors. For example, certain transcription factors known to enhance disease resistance can hinder the expression of cold-tolerance traits, thus negatively impacting the plant’s ability to handle low temperatures [204]. This relationship between genetic expression, pathogen resistance, and abiotic stress tolerance signifies the complex physiological trade-offs plants face in stressful environments.

Because many stress-related traits are polygenic, the integration of genomic tools has become indispensable. Genome-wide association studies (GWAS) and genomic selection approaches can identify loci contributing to stress tolerance, accelerating the improvement of complex traits. For instance, GWAS has successfully identified QTL associated with chilling tolerance in maize [205]. Genetic mapping studies similarly highlight the potential of marker-based prediction and selection for traits with complex inheritance, including drought tolerance [206]. These genomic advances provide a foundation for marker-assisted selection (MAS), which enables breeders to select favorable alleles independently of environmental variation—an advantage particularly relevant under strong GEI [207,208].

The availability of diverse germplasm further strengthens stress-resilience breeding programs. Gene bank resources offer a valuable reservoir of adaptive alleles, highlighting the importance of incorporating broad genetic diversity into breeding pipelines for improved drought and cold tolerance [209].

### 8.3. Genomics-Assisted Breeding (GAB)

Genomics-assisted breeding (GAB) is an effective strategy to improve cold tolerance in legumes by using molecular markers and advanced genomic technologies to identify and incorporate beneficial genes, such as those involved in membrane stability and osmotic adjustment. GAB accelerates the development of climate-smart legume varieties by enabling precise selection of cold-tolerant genotypes and the efficient introgression of cold-tolerance traits into elite cultivars [210,211,212].

The genomics of winter hardiness in legumes focuses on understanding the genetic basis of cold tolerance, which involves complex interactions between genes controlling frost tolerance, cold acclimation, and resistance to biotic and abiotic stresses [172]. Genomic tools like Genome-Wide Association Studies (GWAS), QTL mapping, and marker-assisted selection (MAS) are used to identify specific genetic markers associated with these traits, enabling breeders to select for superior winter-hardy varieties [172,213]. Key genetic mechanisms include the activation of genes like *CBF*, *COR*, and *LEA*, and the modulation of cellular processes such as osmotic stabilization, antioxidant production, and cell wall composition [172,213].

The genomics of winter hardiness in legumes involves identifying genes associated with frost tolerance and other stress responses, using techniques like genome-wide association studies (GWAS) to find genetic markers linked to traits like root health and survival under cold [214]. These studies are used to identify specific genetic regions (SNPs) linked to frost tolerance and other winter hardiness traits. For example, a GWAS on faba bean identified nine novel SNPs associated with root-related traits under frost stress [214]. Key genes, such as those in the C-repeat binding factor family (CBF family) [213], and quantitative trait loci (QTLs) on various chromosomes [214] are known to play a significant role in this complex trait. The QTL approach identifies multiple genes that contribute to a trait. Numerous QTLs have been linked to freezing tolerance in various legumes [213]. Once specific markers are identified, breeders can use them to select for favorable alleles during the breeding process, speeding up the development of new varieties [213]. An advanced haplotype-based breeding method will further allow breeders to assemble desirable combinations of genes to tailor crop varieties for specific traits [213].

Modern breeding strategies leverage advanced genomic resources, high-throughput genotyping and phenotyping [150], and haplotype-based breeding [215] to improve winter-hardy legume varieties more efficiently. These genomic tools pave the way for overcoming breeding challenges. While traditional and genomic breeding have delivered winter-hardy legume cultivars, persistent challenges in field evaluation, genotype x environment interactions, and polygenic control remain.

### 8.4. Major Functional Genes Related to Winter Hardiness

One prominent candidate gene associated with winter hardiness is the CBF/DREB1 transcription factor family, which plays a crucial role in plant response to cold stress. The conservation of genetic control of frost tolerance among legumes indicates that CBF genes are located near frost damage QTLs identified through Genome-Wide Association Studies (GWAS) [216]. This underscores the potential of CBF genes as key targets in the development of winter-hardy legume varieties due to their role in mediating cold tolerance. It was identified that a specific locus on chromosome 1 correlates with frost tolerance in faba bean [217]. This genomic region has been consistently highlighted across different studies as potentially harboring genes that confer winter hardiness, providing strong support for the targeted breeding of faba bean lines that express favorable alleles at this locus.

In the context of alfalfa (*Medicago sativa*), it was reported that the *MsaciB* gene, which is positively correlated with winter hardiness and regrowth traits, further emphasizes its potential utility in breeding programs targeting improved winter performance in legumes [218]. The genetic parallels drawn between alfalfa and faba bean can provide insights into developing robust faba bean varieties through cross-species comparative analysis. Similarly, the fine-mapping identified multiple QTL linked to freezing tolerance in alfalfa, revealing actionable genetic loci on various chromosomes that could inform breeding efforts for winter hardiness [219]. Their findings reinforce the idea that genetic diversity within legumes can be systematically leveraged to improve frost resistance.

Furthermore, linkage mapping and GWAS were applied to identify significant QTL associated with frost tolerance in faba bean, thereby establishing a genetic framework essential for marker-assisted selection [220]. Their findings highlight the potential for precision breeding approaches to incorporate frost resistance traits into commercial faba bean varieties. Faba bean research can benefit from the genomic resources available for *M. truncatula*, which exhibit genetic variation for freezing tolerance and can provide candidate genes for winter hardiness enhancement in faba bean [221].

In addition, the identification of novel alleles within the faba bean genome can yield new opportunities for improving winter resilience. For example, robust mapping of genetic loci in ongoing faba bean research suggests that alleles linked to abiotic stress response—particularly in relation to cold stress—can greatly enhance breeding programs’ efficiency [222]. However, the link between these alleles and direct winter hardiness traits requires further study.

## 9. Challenges and Progress in Breeding for Winter Hardiness in Grain Legumes

Breeding for overwintering ability resulted in significant improvement in chickpea (*Cicer arietinum* L.) genotypes: the seed yield of 19 breeding lines sown in autumn was significantly higher than that sown in spring (3227 and 1600 kg ha^−1^, respectively) [223]. In addition, overwintering ability is a very important characteristic in other aspects, since a high survival rate may result in adequate plant density in spring, leading to sufficient soil coverage and finally to a high yield [13]. Breeding results for abiotic stress tolerance, including winter hardiness, have been collected, and important autumn-sown legumes have been introduced in earlier published review papers and book chapters [21,224,225,226,227].

Under field conditions, the evaluation of winter hardiness is challenging and time-consuming. In faba bean, for example, plant survival is strongly affected not only by genotype but also by year-to-year variation in weather conditions (e.g., daily minimum temperature, duration of snow cover). During a three-year experiment, the first year was suitable for distinguishing cultivars according to their winter hardiness, whereas the hard winter killed almost all faba bean plants in the second year. However, in the third year, a mild winter occurred, which allowed almost all plants to survive [13]. In some crop years, the selection of cold-resistant genotypes may be hindered by the mild winter [228]. The overwintering ability based on the survival percentage can be categorized as follows: (i) excellent: >75%; (ii) good: 50–75%; (iii) marginal: 25–50%; and (iv) poor: < 25% [229].

Although the winter injuries were found to be serious for some winter pea genotypes in experiments established in Turkey, they showed high-rate winter survival due to their ability to recover [126]. Differences in survival rate were detected between pea genotypes: significantly more winter-type plants from the germplasm collection survived during winter compared to local landraces [126]. However, in laboratory conditions, all plants died when −12 and −16 °C treatments were applied to pea genotypes [126]. Although a high correlation (r = 0.68 − 0.87) for survival rate was detected between field and laboratory conditions, field tests suggested performing them during the final selection period because several other factors (e.g., snow cover, soil condition, wind) may influence the survival results in field experiments [126].

The rate of survival was also significantly influenced by the different autumn sowing dates: the earliest (1 October) sowing resulted in the lowest (88.8%) survival compared to those sown later (97.8 and 97.9%, 15 October and 1 November, respectively) [230]. Moreover, interaction between cultivar and crop year was found for winter survival in winter faba bean in experiments conducted in eastern Austria [17]. High survival rates were found in ‘Aviron’, ‘Enduro’, and ‘James’ winter peas (97.9%, 97.4%, and 97.8%, respectively), whereas the Curling cultivar survived at a rate of 89.5% [16]. In the case of faba bean, ‘Diva’ survived at a rate of 93.1%, whereas 89.5% of ‘Hiverna’ plants survived [17]. Some results of field experiments are summarized in Table S1.

Wild relatives, breeding lines, landraces, etc., that can be used as sources for winter hardiness in breeding programs have been grouped by their useful traits in the aspects of breeding aims and have been described for peas, lentils, and chickpeas [231]. The morphological, physiological, and molecular effects of low temperature (chilling and freezing stress) on the growth and development of chickpea and lentil are very well summarized and described in a recently published book chapter [116].

Breeding for drought and heat resistance is the main aim in the development of new varieties in chickpea (*Cicer arietinum* L.) [232]. However, resistance to cold stress and low temperature may also be very important in cultivars for growing regions such as the Mediterranean. In general, chickpeas have relatively good frost tolerance (−8 °C); some genotypes can survive even −12 °C [233]. Significant genotype × environment interaction was found for each character studied in field experiments conducted in five locations in Iran. In the chickpea sown in autumn, the amount of rainfall may have a strong effect on the interaction between the environment and grain yield. One genotype (FLIP 10–128 C) proved to be particularly suitable for autumn sowing, based on its high yield [234].

Winter hardiness, including freezing tolerance, has proven to be a quantitative trait with high heritability [235]. Mass selection was applied in order to increase the winter hardiness of faba bean breeding material up to −25 °C, and populations created by selection showed very various morphological, developmental, and nutritional characteristics [236]. Five genomic regions were identified in the regulation of frost tolerance in faba bean, located on the linkage groups I, III, IV, and V. The quantitative trait loci (QTLs) were associated with frost damage symptoms (*FD1*, *FD2*, *FD3*, and *AUSPC*) and with plant survival (*SR*). Breeding lines with those QTLs showing high frost tolerance and survival ability have been identified and are available for breeding programs [216].

Breeding aims and achievements for horse beans are well summarized in a review [79]; thus, we only processed the relevant literature published after 2010 in this study. Similarly, the factors affecting winter hardiness of alfalfa (*Medicago sativa* L.) and the genetic background of frost tolerance were reviewed in the early 2000s [237]. Being a perennial plant, alfalfa has a certain ability to withstand winter, but increasing winter resistance is one of the breeding goals of this crop as well [238].

Nowadays, in the southern part of Europe, the pea (*Pisum sativum* L.) is sown in autumn (most frequently in the second decade of November) as a winter crop, whereas it is grown as a spring crop in the middle and northern parts of Europe [117]. Since the pea (*Pisum sativum* L.) is a cold-season leguminous plant, it has a certain degree of cold tolerance, but the winter tolerance of most varieties is not very good; therefore, the production of varieties suitable for winter sowing requires serious selection work. The pea’s ability to tolerate frost is limited to the vegetative phase; it is sensitive to frost in the generative phase [239]. Although peas may germinate at a wide range of temperatures from 1.1 to the optimum 20 °C, 50% of seedlings will be killed at −4.5 °C [117]. The cold tolerance test, suitable for testing and selecting different pea genotypes, includes an acclimation or hardening period (temperature gradually decreases during a 3–4-week period), then exposure to low temperatures (up to −9 °C, over 6–8 h) followed by warming to 3 °C (over 6–8 h). It can be evaluated after 2–3 weeks of recovery [240]. Several F5 lines (originated from winter pea collections in the USA, France, and Austria) were identified as genotypes with good winter hardiness and high yielding capacity (5–6 t ha^−1^), combined with proper early maturity [241]. Winter peas can also be sown as a soil cover crop, and 18 breeding lines were tested for their winter hardiness in two sites of the United States (the Southeast and mid-Atlantic regions). In Maryland better results were achieved by growing purple clover and hairy vetch (*Vicia villosa* Roth.), due to serious winter damage of peas. However, the productivity of winter peas was similar to the other two crops in North Carolina [242]. Other newly emerged breeding aims besides winter hardiness are heat and herbicide tolerance/resistance, easier digestibility, and multi-purpose usability of herbicides. In addition to the survival rate, the presence and extent of yellowing and necrosis on vegetative parts, especially on leaves, are the most common evaluation aspects for winter hardiness [239].

Pea plants developed from seeds sown in spring, if they grow under optimal conditions, will be sensitive to the cold (even 2 °C can be harmful) and may be damaged during flowering. However, in the case of autumn sowing, such genotypes gradually get used to the cold and can even survive −9 °C [240]. In the case of peas, winter tolerance should also be distinguished from frost tolerance, as many other factors can be taken into account in addition to cold temperatures. Such factors influencing winter hardiness are, for example, the response to the length of the daily light, the soil conditions (type, water status, microbiome interactions), wind, and soil cover (stubble, snow). The timing and duration of adverse conditions are also of great importance [240].

Polish researchers have developed and validated a versatile PCR marker array that allows the separation of spring and winter-type white lupine ecotypes by molecular selection. Molecular markers (DArT-seq, silicoDArT, and LalbFTc1) were identified and validated, which were associated with the traits responsible for the spring or autumn type of white lupine landraces (flowering time and vernalization responsiveness) [243].

Summarizing, the breeding for overwintering ability requires the improvement of many physiological properties, such as tolerance to low temperature, to frost, to permanent snow cover, to frost-drought, to mechanical soil movement, and to winter disease tolerance [244]. Additionally, in the case of horse beans (faba bean; *Vicia faba* L.), for example, developing the appropriate vernalization requirement (to avoid flowering before winter) also may be involved [244].

### New Varieties Developed for Winter Hardiness

The development of winter legume cultivars has been a major objective in breeding programs across temperate regions, aiming to extend the possibilities of the cultivation range of grain legumes and enhance yield stability through autumn sowing. The winter legume varieties developed for autumn sowing in Europe until 2010 have been reviewed, highlighting the initial breakthroughs in cold-tolerant germplasm development for pea, faba bean, lentil, and chickpea [21]. Since 2010, several winter-hardy cultivars and advanced breeding lines have been registered worldwide, reflecting intensified global efforts to adapt cool-season grain legumes to autumn sowing under variable winter conditions.

In Romania, the first winter pea variety (Lavinia F) was registered recently, which showed very good winter hardiness and produced a high yield (4.2 t ha^−1^). In addition, it has resistance to lodging, to drought, to Erysiphe pisi, and to Ascochyta [245]. The winter pea variety (NS-Mraz), bred in Novi Sad and registered (2011) as the first winter pea variety for grain production in Serbia, shows very good winter tolerance and productivity (5.5 t ha^−1^). It is a very early cultivar, which ripens uniformly and is resistant to lodging [246]. Due to its high frost tolerance (able to survive up to −17 °C), it is often involved in adaptation experiments [247]. Four winter-hardy faba bean germplasm lines (WH-1, WH-2, WH-3, and WH-4) were developed in response to U.S. demand for annual winter legumes. These lines demonstrated an 84% average increase in winter survival, achieving comparable winter hardiness to European materials [248].

Legume breeding has a long tradition at the Nyíregyháza Research Institute, University of Debrecen, and we make continuous efforts to improve the adaptability and productivity of key species such as pea (*Pisum sativum* L.), broad bean (*Vicia faba* L.), lentil (*Lens culinaris* Medik.), bean (*Phaseolus vulgaris* L.), or hairy vetch (*Vicia villosa* Roth.). Over several decades, numerous high-performing cultivars, breeding lines, and pre-breeding populations have been developed and thoroughly evaluated within our program. However, the rapidly changing environmental conditions have shifted the primary breeding focus toward enhancing tolerance to abiotic stresses. In our region, recurrent atmospheric drought, heat waves, and low and unevenly distributed precipitation have become major constraints on legume production. As attention has increasingly turned toward sustainable solutions to these climatic challenges, the breeding of winter-hardy legume species has come into focus. Currently, special emphasis is placed on developing genotypes with enhanced overwintering ability, as the shift toward autumn sowing represents a promising strategy to improve yield potential, enhance biological nitrogen fixation efficiency, and optimize the utilization of early spring soil moisture. As a result of these efforts, the Research Institute has two winter-hardy lentil varieties (‘Pinklevi’ and ‘Rézi’) and one winter pea candidate (‘Réku’). Breeding work is ongoing, and we are launching breeding programs for more legume species to develop winter varieties (e.g., faba beans).

## 10. Future Prospects and Breeding Strategies for Enhanced Winter Hardiness

Increasingly frequent and extreme abiotic stresses such as cold, frost, and winter desiccation, induced by climate change, fundamentally threaten the yield stability of crops, posing a serious challenge to global food security. Targeted breeding of plant varieties with increased winter hardiness is, therefore, a critical strategic task. Overcoming the time-consuming limitations of traditional breeding methods, modern biotechnology and the genomic revolution offer molecular-based approaches that can radically increase the efficiency and accuracy of the breeding process in improving complex, polygenic winter hardiness.

Genomic Selection (GS) is the key to future breeding strategies, which allows the estimation of breeding value (Genomic Estimated Breeding Value, GEBV) based on DNA markers located throughout the genome and their associated phenotypic data. This approach significantly speeds up the breeding cycle, as selection can be carried out at the level of young plants, without having to wait for the phenotypic evaluation of adult plants, which is particularly time-saving in the case of winter hardiness. This is complemented by marker-assisted selection (MAS), which allows the targeted incorporation of identified QTLs (e.g., Vrn and Fr genes) that play a key role in cold tolerance, frost tolerance, and vernalization stress. Furthermore, the use of genome editing technologies such as the CRISPR/Cas system allows precise genetic modifications in gene families that play a fundamental role in the cold stress response, such as the CBF transcription factors, which promises to produce adaptive phenotypes extremely quickly and accurately.

The future of winter legume cultivation holds great potential, and an integrated, multidisciplinary strategy is essential for success. This includes high-throughput phenomics tools, including remote sensing and machine learning, that provide objective and quantitative data on plant responses to cold stress, supporting the fine-tuning of genomic models. Pangenomic research can help breeders uncover the full genetic diversity, identifying valuable winter hardiness alleles in crop wild relatives (CWR) and old landraces that can be incorporated into breeding programs. Finally, breeding should consider genotype–environment (G×E) interactions, integrating them into selection models to create more robust, climate-resilient crops that perform reliably across diverse agroecological zones, ensuring the sustainability and stability of future food production.

The development of winter-hardy legumes will be essential for ensuring stable and sustainable crop production under changing climatic conditions, and progress in this field will rely on the synergistic integration of all available breeding approaches—from traditional selection to advanced genomic and biotechnological tools.

## Figures and Tables

**Table 2:** Comparison of the seed cost of faba bean in Ireland based on the price in 2025.

Cultivar	Quality	Type	Seed Price € per kg *	Seed Demand kg per ha	Seed Cost € per ha
Bobas	Organic	Spring	2.5	150	375
n.a.	Organic	Winter	2.95	150–200	442.5–590
Irena	Traditional	Winter	1.4	200–250	280–350

* Source: Fruit Hill Farm—Ireland’s organic farm and garden specialists.

## Data Availability

Not applicable.

## Supplementary Materials

The following supporting information can be downloaded at https://www.mdpi.com/article/10.3390/life16010017/s1, Table S1: Winter survival and yield advantages of autumn sown grain legumes—Global field performance of different legumes species under diverse climatic conditions; References [249,250,251,252,253,254,255,256,257] are cited in the supplementary materials.
