# Supplementary material for: Rising Demand for Winter Crops Under Climate Change: Breeding for Winter Hardiness in Autumn-Sown Legumes
Source: Life (Basel). 2025 Dec 22;16(1):17. doi: 10.3390/life16010017 (PMC12843293; doi:10.3390/life16010017)
Supplement: Supplementary file 1 [file life-16-00017-s001.zip › life-4014298-supplementary.pdf]

Table S1. Winter survival and yield advantages of autumn-sown grain legumes—Global field performance of different legumes species under diverse climatic conditions

Species/Varieties	Region	Sowing period	Survival (%)	Advantage/Best results	References
**Winter pea**					
Winter pea (*Pisum sativum* L.)<br>Aviron (1),<br>Cherokee (2),<br>Curling (3),<br>Enduro (4),<br>Isard (5),<br>James (6)<br>Spring type: Astronaute	Eastern-Austria	2nd decade in October	97.9 (1)<br>91.1 (2)<br>89.5 (3)<br>97.4 (4)<br>94.8 (5)<br>97.8 (6)<br>mean: 94.6	Dry matter yield of aboveground biomass (AGB) was 1.36–2.3 x higher;<br>Grain yield was 1.93 x higher in the average of cultivars;<br>Pod density was 2.68 x higher;<br>Grain density (grain m^{-2}) was 2.41 x higher<br><br>compared to spring-type Astronaute	[17, 251]
Field pea (ssp. *arvense*) and garden pea (ssp. *sativum*)<br>Landraces (31)<br>Lines (3),<br>Selection (4)<br>Accessions (4)<br>Cultivars (16)<br>Total: 58 genotypes	Turkey<br>Central Anatolia	2nd decade in October	1.5–100%<br>Turkish landraces:<br>84.8–88.6%<br>Control cvs:<br>85–94.7%	More than 90% survival rate:<br>GRU 809 (UK selection)<br>PI392018 (accession from Bulgaria)<br>PI392019 (accession from the Soviet Union)<br>PI517923 (accession from Bulgaria)<br>Melrose (cultivar)<br>Özkaynak (cultivar)<br>Taskent (cultivar)	[126]
Forage pea cultivars Golyazi,<br>Ozkaynak,<br>Taskent,<br>Tore,<br>Urunlu,<br>Ardahan (population)	Turkey<br>Eskişehir<br>(Central Anatolia)<br>terrestrial climate, 800 m ASL	2nd decade in October	na	Fresh forage yield/dry matter content in average of cultivars: spring sowing: 13.42 /3.36 t ha^{-1}; autumn sowing: 24.36/5.73 t ha^{-1};<br>Crude protein content of seeds in average of cultivars: spring sowing: 18.53%; autumn sowing: 21.17%;<br>Best results: Ozkaynak, Taskent, Tore	[118]
Forage pea (*Pisum sativum* var. *arvense* L. Poir.)<br>Töre (1),<br>Özkaynak (2),<br>Taşkent (3),<br>Ulubatlı (4),<br>Ürünlü (5),<br>Gölyazı (6)	Turkey<br>Eskişehir<br>(Central Anatolia)<br>terrestrial climate, 800 m ASL	October 1, October 15, November 1	Töre (1), 98.2<br>Özkaynak (2) 97.0,<br>Taşkent (3) 97.5,<br>Ulubatlı (4) 87.9,<br>Ürünlü (5) 96.5,<br>Gölyazı (6) 91.8<br>October 1: 88.8<br>October 15: 97.8,<br>November 1: 97.9	the best: Töre<br>the worst: Ulubatlı	[231]

Field pea, mostly landraces, from China: 906 winter type, 1411 spring type; and 402 foreign genotypes; and 953 others, a total of 3672 accessions	China, Qingdao, Shandong, 15 m ASL, monsoon-influenced temperate climate	October 20	214 accessions highly resistant (HR) without damage; 835 accessions moderately resistant (MR) genotype: some damages, but able to recover in spring; 2623 accessions susceptible (S) genotype were completely killed.		[252]
Winter (forage) pea NS-Moroz, Enduro Svit (spring type) Darunok stepu (spring type)	Ukraine, Odessa region	October 05 October 15 October 25	na	Svit: fast early growth (18–20 cm), others: 12–15 cm; NS-Moroz: the longest roots (12–15 cm), others: 5–9 cm; ripening 15–20 days earlier than spring-sown plants; optimum: October 15 sowing; best cultivars: NS-Moroz and Enduro	[253]
Forage peas hybrid lines: №PL, №6, №11, №12A, №13, №14, varieties: Taskent, Tore, Mir (control)	Bulgaria, Danube hilly plain region	2nd decade in October	na	Vegetation period (days): Tore: 240.5; Taskent: 243.5; №14: 226.5; №13: 240.5; №12A: 233; №11: 239.5; №6: 235; №PL: 237; Mir: 233.5 The grain yield of the hybrids exceeded the control (Mir) yield by 12.1–43.2%; the best lines were №14, №PL, and №12A	[127]
Winter pea (*Pisum sativum* L.) NS-Moroz (syn. NS-Mraz) Partner Kosmaj Shal (spring type, control)	South-east part of Kazakhstan arid foothill zone, continental climate	n.a.	NS-Moroz: 86% Partner: 30% Kosmaj: 89%	Ripening: NS-Moroz: June 20; Partner: June 20; Kosmaj: n.a.; Shal: July 05 Yield: NS-Moroz: 37.2 c ha^{-1}; Partner: 12.9 c ha^{-1}; Kosmaj: 267.7 c ha^{-1} green mass; Shal: 13.3 c ha^{-1}	[249]
Winter pea (*Pisum sativum* L.) breeding lines PS3057M1, PS3057M2, PS3073G1, PS3073G2, PS3073G3, PS4028H1, PS4028H2, PS4028H3, PS4028H4, PS4053M1, PS4053M2, PS4053M3, PS4053M4 and control varieties: Emirbey, Şahin	Turkey, Konya, 1020 m ASL, continental climate	2nd decade in October	no winter damage	Seed yield (t ha^{-1}): PS3057M1: 1.88; PS3057M2: 2.81; PS3073G1: 2.1; PS3073G2: 2.06; PS3073G3: 1.82; PS4028H1: 2.55; PS4028H2: 2.71; PS4028H3: 2.73; PS4028H4: 2.76; PS4053M1: 2.02; PS4053M2: 2.14; PS4053M3: 1.71; PS4053M4: 2.84; Emirbey: 2.6; Şahin: 2.64	[114]
Austrian winter pea, as a cover crop Arvica 4010 (1) Dunn (2) Frost Master (3) Lynx (4) Maxum (5)	Coffeeville, United States	2nd decade in October	two crop years: 25–38 (1) 25 (2) 89–96 (3) 47–87 (4)		[230]

Survivor 15 (6)<br>Whistler (7)<br>Windham (8)			25–45 (5)<br>70–95 (6)<br>69–100 (7)<br>69–100 (8)		
**Faba bean**					
Winter faba bean (*Vicia faba* L.)<br>Arthur (1),<br>Sultan (2),<br>Hiverna (3),<br>Husky (4),<br>Diva (5),<br>Diver (6),<br>Gladice (7),<br>Alameda (8)	Eastern-Austria	2nd decade in October	2010/2011<br><50.0 (1)<br><50.0 (2)<br>73.0 (3)<br>53.0 (4)<br>76.0 (5)<br><50.0 (6)<br><50.0 (7)<br>0 (8)	16–79% more shoots per plant in winter types<br>TGW 388–471 g in winter types, spring type: Alexia (298 g)<br>Grain yield per shoot was higher in Arthur, Husky, and Gladice<br>Grain yield per pod was higher in Sultan, Husky, and Gladice<br>Diva (5) grain yield higher than spring type	[13]
Winter faba bean (*Vicia faba* L.)<br>Diva (1),<br>Hiverna (2)	Eastern-Austria	2nd decade in October	93.1 (1)<br>89.5 (2)	Dry matter yield of aboveground biomass and grain yield were higher in the average of genotypes:<br>1.56–1.62 x; 1.14–1.4 x<br>compared spring types	[17]
**Lentil**					
Lentil (*Lens culinaris* Medik)<br>Samos (G1)<br>Dimitra (G2)<br>Thessalia (G3)<br>Elpida (G4)<br>03-04L (G5)	Greece, semiarid Mediterranean<br>L5: Domokos (500 m)	L5: end of November	Samos (G1)<br>Dimitra (G2)<br>Thessalia (G3)<br>Elpida (G4)<br>03-04L (G5)	Best genotype:<br>L5: Dimitra (G2) 0.97 t ha^{-1} NS	[254]
Winter lentil (*Lens culinaris* Medik. subsp. culinaris)<br>11 cultivars<br>89 RILs from 'Precoz ' × 'WA8649041' (P×W);<br>118 RILs from 'BGE016365' × 'ILL1918' (B×I);<br>'ILL4400' (Syrian landrace);<br>'Águeda',<br>'Alcor', 'Aljama', 'Amaya', 'Ángela', 'Azagala', 'Candela', 'Guareña', 'Lyda', 'Magda' and 'Paula (cutivars)	Spain, (Valladolid province),<br>(1) Zamadueñas (690 m)<br>(2) Peñaflor de Hornija (839 m)	First half of November (winter sowing);<br>Second half of February (spring sowing)		The yield increase of autumn-sown plants was 111% on average of genotypes compared to those sown in spring.<br>W-33, W-40 and B-79, B-117, B-137 RILs were the best for both sowing times	[255]
Lentil (*Lens culinaris* Medik. subsp. culinaris)<br>253 genotypes	Iran,<br>Mashhad (arid cold temperate region, 985 m)	n.a.	0–100%<br>23 genotypes: >75%;<br>the best: 100%: MLC8	Best results:<br>MLC415: grain yield 88 g m^{-2}, biomass yield 535 g m^{-2}<br>MLC291: plant length: 41 cm;	[229]

	(mild winter:<br>-5.9°C)			MLC218: 83 filled pods per plant	
Lentil (*Lens culinaris* Medik. subsp. culinaris)<br>253 genotypes	Iran,<br>Mashhad (arid cold temperate region, 985 m)<br>(Cold winter:<br>-13°C)	n.a.	0–100%<br>the best: 100%: MLC13	Best results:<br>MLC409: plant length: 53 cm;<br>Freezing-tolerant genotype (yield g m^{-1})<br>MLC424 (154)<br>MLC286 (192)<br>MLC409 (220)<br>MLC303 (144)<br>MLC334 (225)<br>MLC407 (264)	[256]
Lentil (*Lens culinaris* Medik. subsp. culinaris)	Iran,<br>Mashhad (arid cold temperate region, 985 m)<br>Jolgeh Rokh (arid cold region, 1710 m; −12.4 °C)		Mashhad: each 100%<br>Jolgeh Rokh:<br>Best: MLC103: 45.2%	Best results:<br>Biological and grain yield:<br>Mashhad: MLC33<br>Jolgeh Rokh: MLC70<br>MLC409 and MLC70 recommended for cold region	[257]
Lentil (*Lens culinaris* Medik. subsp. culinaris<br>40 genotypes	Mashhad (arid cold temperate region, 985 m)<br>(−12.4 °C)		53 and 92%	Best: (92% survival) MLC8, MLC33<br>Survival and grain yield: MLC8, MLC12, MLC13, MLC33, MLC74, MLC103, MLC469 and MLC742)	[258]
		**Chickpea**			
Chickpea, (*Cicer arietinum* L.)<br>30 desi-type, control:<br>'Saral' (cold-tolerant)<br>MCC505 (cold-sensitive)	Iran,<br>Mashhad (arid cold temperate region, 985 m)<br>Jolgeh Rokh (arid cold region, 1710 m)	2nd decade in October	Mashhad:<br>84–100%<br>'Saral': 100%<br>MCC505: 98%<br>Jolgeh Rokh:<br>0–100%<br>'Saral': 10%<br>MCC505: 0%<br>MCC207: 100%<br>MCC212: 96%<br>MCC291: 89%<br>MCC605: 93%<br>MCC913: 100%<br>MCC259:<br>MCC607:	Superior genotypes: MCC259, MCC607 in cold temperate region, and MCC212 in cold region: taller plants, higher grain yield compared to the other genotypes	[259]
Chickpea (*Cicer arietinum* L.)<br>15 inbred lines<br>'Ana' and 'Nosrat': cold-resistant check genotypes	Locations in Iran: (>1500 m)<br>Maragheh,<br>Zanjan,<br>Hamadan,<br>Kurdistan,<br>Urmia	December		The best genotypes:<br>FLIP 10–128 C, FLIP 10–169 C, and Nosrat (based on their stability and grain yield)	[235]

Abbreviations: ASL: above sea level; na: not available;.NS: non-significant; RIL: recombinant inbred lines
